# Supplementary material for: Impact on childhood mortality of interventions to improve drinking water, sanitation, and hygiene (WASH) to households: Systematic review and meta-analysis
Source: PLoS Med. 2023 Apr 20;20(4):e1004215. doi: 10.1371/journal.pmed.1004215 (PMC10118100; doi:10.1371/journal.pmed.1004215)
Supplement: S1 Annex — Table A. The global burden of infectious disease is mainly due to mortality (per 100,000). Table B. Global Burden of Disease: A moving target? Table C. Description of studies included in this systematic review by WASH technology. Table D. Meta-regression analysis of diarrhoea mortality in childhood by WASH intervention technology. Fig A. All-cause mortality. Fig B. All-cause mortality: Water supply interventions. Fig C. All-cause mortality: Sanitation interventions. Fig D. All-cause mortality: Hygiene interventions. Fig E. All-cause mortality: Drinking water treatment and storage interventions. Fig F. Diarrhoea mortality. Fig G. Effects on all-cause mortality for participants aged over 5 years. Fig H. Funnel graphs with regression lines: All-cause mortality. Fig I. Funnel graphs with regression lines: Diarrhoea mortality. Fig J. Effects on all-cause mortality for nonrandomised studies of interventions at “high risk of bias.” Fig K. Effects on all-cause mortality for randomised controlled trials at “high risk of bias.” (DOCX) [file pmed.1004215.s002.docx]

# S1 Annex

Table A. The global burden of infectious disease is mainly due to mortality (per 100,000)

| *Cause* | *Years of life lost* | *Years living with disability* |
| --- | --- | --- |
| Acute respiratory infection | 1,300 | 10 |
| Diarrhoea | 960 | 100 |

Sources: [1], [2].

*Table B. Global Burden of Disease: a moving target?*

| *Study* | *Affiliation* | *WASH-related deaths* | *95% confidence interval* | |
| --- | --- | --- | --- | --- |
| Ezzati et al. (2002) [3] | IHME | 950,000 |  |  |
| Lim et al. (2012) [4] | IHME | 340,000 | 10,000 | 650,000 |
| Prüss-Üstün et al. (2014) [5] | WHO | 840,000 |  |  |
| Forouzanfar et al. (2015) [6] | IHME | 1,400,000 | 1,240,000 | 1,580,000 |
| Prüss-Üstün et al. (2019) [7] | WHO | 830,000 | 750,000 | 900,000 |
| Murray et al. (2020) [8] | IHME | 850,000 |  |  |

Notes: figures rounded to the nearest 10,000. Global deaths from infectious disease attributable to WASH, according to Institute of Health Metrics and Evaluation (IHME) and the WHO.

*Table C. Description of studies included in this systematic review by WASH technology*

| *Study by main WASH technology* | *Country* | *Location* | *WASH technology* | *Intervention* | *Age groups* | *Baseline water* | *Baseline sanitation* | *Design* | *N* | *Outcome* | |
| --- | --- | --- | --- | --- | --- | --- | --- | --- | --- | --- | --- |
| **Water supply improvement** | | | | | | | | | | |  |
| Abou-Ali et al. [9] | Egypt | National | Piped water supply | Decentralisation (CDD) | 0-59s | Improved | Unimproved | NRSI (matched cross-section) | 372 | All-cause mortality | |
| Ercumen et al. [10]* | India | Urban | Piped water supply | Direct hardware provision | 0-23s | Improved | Improved | NRSI (matched cohort) | 3,408 | All-cause mortality | |
| Ryder et al. [11] | Panama | Rural | Piped water supply | Direct hardware provision | 0-59s | Unimproved | Unimproved | NRSI (natural experiment cohort design) | 192 | Diarrhoea mortality | |
| **Drinking water treatment and storage** | | | | | | | | | | |  |
| Boisson et al. [12] | DRC | Rural | Household water treatment provision (LifeStraw filter) | Direct hardware provision | 0-59s | Unimproved | Unimproved | RCT | 1,144 | All-cause mortality | |
| Conroy et al. [13] | Kenya | Rural | Solar disinfection (SODIS) | Direct hardware provision | 0-71s | Unimproved | Unimproved | Quasi-RCT | 349 | All-cause mortality | |
| Crump et al. [14] | Kenya | Rural | Household water treatment provision (flocculant) | Direct hardware provision | 0-59s; all ages | Unimproved | Unimproved | Cluster-RCT | 363 (0-59s); 3,262 | All-cause mortality | |
| Crump et al. [14] | Kenya | Rural | Household water treatment provision (chlorine) | Direct hardware provision | 0-59s; all ages | Unimproved | Unimproved | Cluster-RCT | 352 (0-59s); 3,388 | All-cause mortality | |
| Du Preez et al. [15] | Kenya | Rural and urban | Solar disinfection (SODIS) | Direct hardware provision | 6-59s | Unimproved | Unimproved | RCT | 1,089 | All-cause mortality | |
| Ercumen et al. [16]* | Bangladesh | Rural | Household water treatment (chlorine) and safe storage | Direct hardware provision | 6-30s | Improved | Unimproved | RCT | 1,814 | All-cause mortality | |
| Ercumen et al. [16]* | Bangladesh | Rural | Safe storage | Direct hardware provision | 6-30s | Improved | Unimproved | RCT | 906 | All-cause mortality | |
| Jain et al. [17] | Ghana | Urban | Household water treatment provision (chlorine) | Direct hardware provision | All ages | Unimproved | Unimproved | RCT | 3,240 | All-cause mortality | |
| Luby et al. [18] | Pakistan | Urban | Household water treatment provision (chlorine) | Direct hardware provision | All ages | Unimproved | Improved | Cluster-RCT | 2,245 | All-cause mortality | |
| Luby et al. [18] | Pakistan | Urban | Household water treatment provision (flocculant) | Direct hardware provision | All ages | Unimproved | Improved | Cluster-RCT | 2,338 | All-cause mortality | |
| Luby et al. [19] | Bangladesh | Rural | Household water treatment provision (chlorine) | Direct hardware provision | 0-23s | Improved | Unimproved | Cluster-RCT | 1,781 | All-cause mortality | |
| Lule et al. [20] | Uganda | Rural | Household water treatment (chlorine) and safe storage | Direct hardware provision | All ages | Unimproved | Unimproved | RCT | 2,201 | All-cause mortality | |
| Mengistie et al. [21] | Ethiopia | Rural | Household water treatment (chlorine) | Direct hardware provision | 0-59s | Unimproved | Unimproved | Cluster-RCT | 845 | All-cause mortality | |
| Morris et al. [22] | Kenya | Rural | Household water treatment (filter) | Direct hardware provision | 4-16s | Unimproved | Unimproved | RCT | 240 | All-cause mortality | |
| Null et al. [23] | Kenya | Rural | Household water treatment (chlorine) | Direct hardware provision | 0-23s | Unimproved | Unimproved | Cluster-RCT | 1,362 | All-cause mortality | |
| Peletz et al. [24] | Zambia | Rural | Household water treatment (Lifestraw filter) and container | Direct hardware provision | 0-23s | Unimproved | Unimproved | RCT | 120 | All-cause mortality; diarrhoea mortality | |
| **Sanitation improvement** | | | | | | | | | | |  |
| Emerson et al. [25] | Gambia | Rural | Latrine provision | Direct hardware provision | All ages | Unimproved | Unimproved | Cluster-RCT | 4,836 | All-cause mortality | |
| Gebre et al. [26] | Ethiopia | Rural | Sanitation (latrine slab provision, latrine promotion) | Direct hardware provision with health messaging | 12-59s; all ages | Unimproved | Unimproved | Cluster-RCT | 5,507 (12-59); 35,052 | All-cause mortality | |
| Luby et al. [19] | Bangladesh | Rural | Latrine provision | Direct hardware provision | 0-23s | Improved | Unimproved | Cluster-RCT | 1,740 | All-cause mortality | |
| Null et al. [23] | Kenya | Rural | Sanitation (latrine provision and potties) | Direct hardware provision | 0-23s | Unimproved | Unimproved | Cluster-RCT | 1,348 | All-cause mortality | |
| **Hygiene improvement** | | | | | | | | | | |  |
| Bowen et al. [27] | Pakistan | Urban | Soap | Direct hardware provision with health messaging | 0-95s | Unimproved | Improved | Cluster-RCT | 526 | All-cause mortality; diarrhoea mortality | |
| Cole et al. [28] | South Africa (informal housing) | Urban | Soap, detergent and health education | Direct hardware provision with health messaging | 0-59s | Unimproved | Unimproved | NRS (non-randomized cluster trial) | 470 | All-cause mortality | |
| Cole et al. [28] | South Africa (formal housing) | Urban | Soap, detergent and health education | Direct hardware provision with health messaging | 0-59s | Improved | Unimproved | NRS (non-randomized cluster trial) | 406 | All-cause mortality | |
| Gyorkos et al. [29] | Peru | Urban | Hygiene education | Health messaging | 120s (10-year-olds) | Unimproved | Unimproved | Cluster-RCT | 1,285 | All-cause mortality | |
| Luby et al. [30] | Pakistan | Urban | Antibacterial soap provision | Direct hardware provision with health messaging | 0-35s | Unimproved | Improved | Cluster-RCT | 2,358 | All-cause mortality | |
| Luby et al. [30] | Pakistan | Urban | Plain soap provision | Direct hardware provision with health messaging | 0-35s | Unimproved | Improved | Cluster-RCT | 2,468 | All-cause mortality | |
| Luby et al. [18] | Pakistan | Urban | Soap provision | Direct hardware provision with health messaging | All ages | Unimproved | Improved | Cluster-RCT | 2,207 | All-cause mortality | |
| Luby et al. [19] | Bangladesh | Rural | Handwashing station and soap provision | Direct hardware provision with health messaging | 0-23s | Improved | Unimproved | Cluster-RCT | 1,041 | All-cause mortality | |
| Nicholson et al. [31] | India | Urban | Soap provision | Direct hardware provision with social marketing | 60-71s | Improved | Unimproved | Cluster-RCT | 2,155 | All-cause mortality | |
| Null et al. [23] | Kenya | Rural | Handwashing station and soap provision | Direct hardware provision with health messaging | 0-23s | Unimproved | Unimproved | Cluster-RCT | 1,377 | All-cause mortality | |
| Ram et al. [32] | Bangladesh | Rural | Handwashing station and promotion | Direct hardware provision with social marketing | 0-1s | Improved | Unimproved | RCT | 253 | All-cause mortality | |
| Rhee et al. [33] | Nepal | Rural | Handwashing with soap and water | Direct hardware provision with health messaging | 0-1s | Improved | Unimproved | NRSI (cohort design) | 23,662 | All-cause mortality | |
| **Multiple WASH improvements** | | | | | | | | | | |  |
| Bowen et al. [27] | Pakistan | Urban | Hygiene promotion and household water treatment | Direct hardware provision with health messaging | 0-95s | Unimproved | Improved | Cluster-RCT | 272 | All-cause mortality | |
| Clasen et al. [34] | India | Rural | Sanitation and hygiene promotion | Promotion (CLTS) and subsidy with hygiene promotion | 0-59s; all ages | Improved | Unimproved | Cluster-RCT | 3,880 (0-59s); 20,283 | All-cause mortality | |
| Galiani et al. [35] | Argentina | Urban | Piped water supply and sewer connection | Privatisation | 0-59s | Improved | Unimproved | NRSI (natural experiment double-difference) | 3,870 | All-cause mortality; mortality due to infection | |
| Granados and Sańchez [36] | Colombia | National | Water supply and sewer connection | Privatisation, small-scale provider | 0-11s | Improved | Improved | NRSI (matched repeated cross-section) | 6,435 | All-cause mortality | |
| Humphrey et al. [37] | Zimbabwe | Rural | Chlorine, latrine and handwashing stations, with hygiene promotion | Direct hardware provision with hygiene counselling | 0-18s | Improved | Unimproved | Cluster-RCT | 1,956 | All-cause mortality | |
| Instituto Apoyo [38] | Honduras | Rural | Water supply, latrines and sewer connection | Decentralisation (CDD) | 0-59s | Improved | Improved | NRSI (matched pipeline) | 1,105 | All-cause mortality | |
| Luby et al. [18] | Pakistan | Urban | Household water treatment (flocculant) and soap provision | Direct hardware provision with health messaging | All ages | Unimproved | Improved | Cluster-RCT | 2,312 | All-cause mortality | |
| Luby et al. [19] | Bangladesh | Rural | Household water treatment, latrines and handwashing promotion | Direct hardware provision with health messaging | 0-23s | Improved | Unimproved | Cluster-RCT | 1,053 | All-cause mortality | |
| Messou et al. [39] | Côte d’Ivoire | Rural | Source water supply, latrines and handwashing promotion | Direct hardware provision with health messaging | 0-59s | Unimproved | Unimproved | NRSI (non-RCT) | 419 | Diarrhoea mortality | |
| Null et al. [23] | Kenya | Rural | Household water treatment, sanitation and handwashing promotion | Direct hardware provision with health messaging | 0-23s | Unimproved | Unimproved | Cluster-RCT | 1,367 | All-cause mortality | |
| Pickering et al. [40] | Mali | Rural | Sanitation promotion (CLTS) and hygiene promotion | Promotion (CLTS) with health messaging | 0-59s; all ages | Unimproved | Unimproved | Cluster-RCT | 3,984 (0-59s); 30,467 | All-cause mortality | |
| Rasella [41] | Brazil | Urban | Water supply and sanitation | Direct hardware provision | 0-59s | Improved | Improved | NRSI (non-RCT) | 892 | All-cause mortality; diarrhoea mortality | |
| Reese et al. [42] | India | Rural | Piped water supply, latrines and handwashing promotion | Direct provision with sanitation and hygiene promotion | 0-59s; all ages | Unimproved | Unimproved | NRSI (cohort design) | 3,301 (0-59s); 13,752 | All-cause mortality | |
| Semenza et al. [43] | Uzbekistan | Urban | Household water treatment (chlorine), safe storage and food hygiene education | Direct provision with health messaging | 0-59s | Unimproved | Improved | RCT | 170 | Diarrhoea mortality | |

Note: * the papers by Ercumen and colleagues are identified in forest plots contained in this supplementary figure as Ercumen et al. (2015a) [16] and Ercumen et al. (2015b) [10].

*Table D. Meta-regression analysis of diarrhoea mortality in childhood by WASH intervention technology*

|  | (1) | | | | (2) | | | | (3) | | | |
| --- | --- | --- | --- | --- | --- | --- | --- | --- | --- | --- | --- | --- |
|  | OR | P>t | 95%CI | | OR | P>t | 95%CI | | OR | P>t | 95%CI | |
| *Panel 1: Intervention technology* |  |  |  | |  |  |  | |  |  |  | |
| 1=Water treatment | 0.25 | 0.19 | 0.03 | 2.31 |  |  |  |  |  |  |  |  |
| 1=Hygiene and improved water supply |  |  |  |  | 0.31 | 0.08 | 0.08 | 1.19 |  |  |  |  |
| 1=Community-wide sanitation and hygiene |  |  |  |  |  |  |  |  | 0.42 | 0.05 | 0.18 | 0.98 |
| Constant | 0.59 | 0.05 | 0.36 | 0.99 | 0.67 | 0.05 | 0.45 | 1.00 | 0.74 | 0.07 | 0.53 | 1.03 |
| *Panel 2: Test information* |  |  |  |  |  |  |  |  |  |  |  |  |
| Number of observations | 10 |  |  |  | 10 |  |  |  | 10 |  |  |  |
| Tau-squared | 0.13 |  |  |  | 0.04 |  |  |  | 0.01 |  |  |  |
| I-squared | 38% |  |  |  | 21% |  |  |  | 10% |  |  |  |
| Adjusted R-squared | 24% |  |  |  | 77% |  |  |  | 92% |  |  |  |

*Fig A. All-cause mortality*

*Fig B. All-cause mortality: water supply interventions*

*Fig C. All-cause mortality: sanitation interventions*

*Fig D. All-cause mortality: hygiene interventions*

*Fig E. All-cause mortality: drinking water treatment and storage interventions*

*Fig F. Diarrhoea mortality*

*Fig G. Effects on all-cause mortality for participants aged over 5 years*

*Fig H. Funnel graphs with regression lines: all-cause mortality*

**

*Fig I. Funnel graphs with regression lines: diarrhoea mortality*

**

*Explanatory note to Figs H-I: Tests for small-study effects assume that there are weaker incentives for researchers and journals to publish smaller sample studies that do not show significant findings. In the Figure, the contour-enhanced funnel graphs* [44] *are overlain with regression lines. Noting that the funnel graph axes are swapped, and y-axis inverted, they indicate that studies are generally symmetrically distributed around the null effect, as confirmed by near-zero (and statistically insignificant) intercept coefficients on the regressions of the effect size on its standard error for all-cause mortality (p>0.62) and for diarrhoea mortality (p>0.25).*

*Fig J. Effects on all-cause mortality for non-randomised studies of interventions at ‘high risk of bias’*

**

*Fig K. Effects on all-cause mortality for randomised controlled trials at ‘high risk of bias’*

*Explanatory note to Figs J-K: risk-of-bias assessment concerns the possible bias of the study in measuring the effect of the WASH intervention on mortality in children aged under 5 years only.*

**References**

[1] M. Naghavi *et al.*, ‘Global, regional, and national age-sex specific mortality for 264 causes of death, 1980–2016: a systematic analysis for the Global Burden of Disease Study 2016’, *The lancet*, vol. 390, no. 10100, pp. 1151–1210, 2017.

[2] T. Vos *et al.*, ‘Global, regional, and national incidence, prevalence, and years lived with disability for 328 diseases and injuries for 195 countries, 1990–2016: a systematic analysis for the Global Burden of Disease Study 2016’, *The Lancet*, vol. 390, no. 10100, pp. 1211–1259, 2017.

[3] M. Ezzati, A. D. Lopez, A. Rodgers, S. Vander Hoorn, and C. J. Murray, ‘Selected major risk factors and global and regional burden of disease’, *The Lancet*, vol. 360, no. 9343, pp. 1347–1360, Nov. 2002, doi: 10.1016/S0140-6736(02)11403-6.

[4] S. S. Lim *et al.*, ‘A comparative risk assessment of burden of disease and injury attributable to 67 risk factors and risk factor clusters in 21 regions, 1990–2010: a systematic analysis for the Global Burden of Disease Study 2010’, *The Lancet*, vol. 380, no. 9859, pp. 2224–2260, 2012.

[5] A. Prüss-Ustün *et al.*, ‘Burden of disease from inadequate water, sanitation and hygiene in low-and middle-income settings: a retrospective analysis of data from 145 countries’, *Trop. Med. Int. Health*, vol. 19, no. 8, pp. 894–905, 2014.

[6] M. Forouzanfar, A. Alexander, H. Anderson, and et al. for the GBD 2013 Risk Factors Collaborators, ‘Global, regional, and national comparative risk assessment of 79 behavioural, environmental and occupational, and metabolic risks or clusters of risks in 188 countries, 1990–2013: a systematic analysis for the Global Burden of Disease Study 2013’, *Lancet Lond. Engl.*, vol. 386, no. 10010, pp. 2287–2323, Dec. 2015, doi: 10.1016/S0140-6736(15)00128-2.

[7] A. Prüss-Ustün *et al.*, ‘Burden of disease from inadequate water, sanitation and hygiene for selected adverse health outcomes: An updated analysis with a focus on low- and middle-income countries’, *Int. J. Hyg. Environ. Health*, vol. 222, no. 5, pp. 765–777, Jun. 2019, doi: 10.1016/j.ijheh.2019.05.004.

[8] C. J. L. Murray *et al.*, ‘Global burden of 87 risk factors in 204 countries and territories, 1990–2019: a systematic analysis for the Global Burden of Disease Study 2019’, *The Lancet*, vol. 396, no. 10258, pp. 1223–1249, Oct. 2020, doi: 10.1016/S0140-6736(20)30752-2.

[9] H. Abou-Ali, H. El-Azony, H. El-Laithy, J. Haughton, and S. Khandker, ‘Evaluating the impact of Egyptian social fund for development programmes’, *J. Dev. Eff.*, vol. 2, no. 4, pp. 521–555, 2010.

[10] A. Ercumen *et al.*, ‘Upgrading a piped water supply from intermittent to continuous delivery and association with waterborne illness: a matched cohort study in urban India’, *PLoS Med.*, vol. 12, no. 10, p. e1001892, 2015.

[11] R. Ryder *et al.*, ‘The childhood health effects of an improved water supply system on a remote Panamanian island’, *Am J Trop Med Hyg*, vol. 34, no. 5, pp. 021–924, 1985.

[12] S. Boisson, M. Kiyombo, L. Sthreshley, S. Tumba, J. Makambo, and T. Clasen, ‘Field Assessment of a Novel Household-Based Water Filtration Device: A Randomised, Placebo-Controlled Trial in the Democratic Republic of Congo’, *PLOS ONE*, vol. 5, no. 9, p. e12613, Sep. 2010, doi: 10.1371/journal.pone.0012613.

[13] R. M. Conroy, M. E. Meegan, T. Joyce, K. McGuigan, and J. Barnes, ‘Solar disinfection of water reduces diarrhoeal disease: an update’, *Arch. Dis. Child.*, vol. 81, no. 4, pp. 337–338, 1999.

[14] J. A. Crump *et al.*, ‘Household based treatment of drinking water with flocculant-disinfectant for preventing diarrhoea in areas with turbid source water in rural western Kenya: cluster randomised controlled trial’, *Bmj*, vol. 331, no. 7515, p. 478, 2005.

[15] M. du Preez *et al.*, ‘Randomized intervention study of solar disinfection of drinking water in the prevention of dysentery in Kenyan children aged under 5 years’, *Environ. Sci. Technol.*, vol. 45, no. 21, pp. 9315–9323, 2011.

[16] A. Ercumen, A. M. Naser, L. Unicomb, B. F. Arnold, J. M. J. Colford, and S. P. Luby, ‘Effects of Source versus Household Contamination of Tubewell Water on Child Diarrhea in Rural Bangladesh: A Randomized Controlled Trial’, *PLOS ONE*, vol. 10, no. 3, p. e0121907, Mar. 2015, doi: 10.1371/journal.pone.0121907.

[17] S. Jain *et al.*, ‘Sodium dichloroisocyanurate tablets for routine treatment of household drinking water in periurban Ghana: a randomized controlled trial’, *Am. J. Trop. Med. Hyg.*, vol. 82, no. 1, p. 16, 2010.

[18] S. P. Luby *et al.*, ‘Combining drinking water treatment and hand washing for diarrhoea prevention, a cluster randomised controlled trial’, *Trop. Med. Int. Health*, vol. 11, no. 4, pp. 479–489, 2006.

[19] S. P. Luby *et al.*, ‘Effects of water quality, sanitation, handwashing, and nutritional interventions on diarrhoea and child growth in rural Bangladesh: a cluster randomised controlled trial’, *Lancet Glob. Health*, vol. 6, no. 3, pp. e302–e315, 2018.

[20] J. R. Lule *et al.*, ‘Effect of home based water chlorination and safe storage on diarrhea among persons with human immonodeficiency virus in Uganda.’, *Am. J. Trop. Med. Hyg.*, vol. 73, no. 5, pp. 926–933, 2005.

[21] B. Mengistie, Y. Berhane, and A. Worku, ‘Household Water Chlorination Reduces Incidence of Diarrhea among Under-Five Children in Rural Ethiopia: A Cluster Randomized Controlled Trial’, *PLOS ONE*, vol. 8, no. 10, p. e77887, Oct. 2013, doi: 10.1371/journal.pone.0077887.

[22] J. F. Morris *et al.*, ‘A Randomized Controlled Trial to Assess the Impact of Ceramic Water Filters on Prevention of Diarrhea and Cryptosporidiosis in Infants and Young Children—Western Kenya, 2013’, *Am. J. Trop. Med. Hyg.*, vol. 98, no. 5, pp. 1260–1268, May 2018, doi: 10.4269/ajtmh.17-0731.

[23] C. Null *et al.*, ‘Effects of water quality, sanitation, handwashing, and nutritional interventions on diarrhoea and child growth in rural Kenya: a cluster-randomised controlled trial’, *Lancet Glob. Health*, vol. 6, no. 3, pp. e316–e329, Mar. 2018, doi: 10.1016/S2214-109X(18)30005-6.

[24] R. Peletz *et al.*, ‘Assessing water filtration and safe storage in households with young children of HIV-positive mothers: a randomized, controlled trial in Zambia’, *Am. J. Trop. Med. Hyg.*, vol. 101, no. 3, pp. 555–565, 2012, doi: 10.4269/ajtmh.18-0888.

[25] P. M. Emerson *et al.*, ‘Role of flies and provision of latrines in trachoma control: cluster-randomised controlled trial’, *The Lancet*, vol. 363, no. 9415, pp. 1093–1098, 2004.

[26] T. Gebre *et al.*, ‘Latrine Promotion for Trachoma: Assessment of Mortality from a Cluster-Randomized Trial in Ethiopia’, *Am. J. Trop. Med. Hyg.*, vol. 85, no. 3, pp. 518–523, Sep. 2011, doi: 10.4269/ajtmh.2011.10-0720.

[27] A. Bowen, M. Agboatwalla, S. Luby, T. Tobery, T. Ayers, and R. M. Hoekstra, ‘Association between intensive handwashing promotion and child development in Karachi, Pakistan: a cluster randomized controlled trial’, *Arch. Pediatr. Adolesc. Med.*, vol. 166, no. 11, pp. 1037–1044, 2012.

[28] E. C. Cole *et al.*, ‘Comprehensive family hygiene promotion in peri-urban Cape Town: Gastrointestinal and respiratory illness and skin infection reduction in children aged under 5’, *South Afr. J. Child Health*, vol. 6, no. 4, pp. 109–117, 2012.

[29] T. W. Gyorkos, M. Maheu-Giroux, B. Blouin, and M. Casapia, ‘Impact of health education on soil-transmitted helminth infections in schoolchildren of the Peruvian Amazon: a cluster-randomized controlled trial’, *PLoS Negl. Trop. Dis.*, vol. 7, no. 9, p. e2397, 2013.

[30] S. P. Luby, M. Agboatwalla, J. Painter, A. Altaf, W. L. Billhimer, and R. M. Hoekstra, ‘Effect of Intensive Handwashing Promotion on Childhood Diarrhea in High-Risk Communities in Pakistan: A Randomized Controlled Trial’, *JAMA*, vol. 291, no. 21, pp. 2547–2554, Jun. 2004, doi: 10.1001/jama.291.21.2547.

[31] J. A. Nicholson *et al.*, ‘An investigation of the effects of a hand washing intervention on health outcomes and school absence using a randomised trial in Indian urban communities’, *Trop. Med. Int. Health*, vol. 19, no. 3, pp. 284–292, 2014, doi: 10.1111/tmi.12254.

[32] P. K. Ram *et al.*, ‘Impact of an Intensive Perinatal Handwashing Promotion Intervention on Maternal Handwashing Behavior in the Neonatal Period: Findings from a Randomized Controlled Trial in Rural Bangladesh’, *BioMed Res. Int.*, 2017, doi: Doi:10.1155/2017/6081470.

[33] V. Rhee *et al.*, ‘Maternal and Birth Attendant Hand Washing and Neonatal Mortality in Southern Nepal’, *Arch. Pediatr. Adolesc. Med.*, vol. 162, no. 7, pp. 603–608, Jul. 2008, doi: 10.1001/archpedi.162.7.603.

[34] T. Clasen *et al.*, ‘Effectiveness of a rural sanitation programme on diarrhoea, soil-transmitted helminth infection, and child malnutrition in Odisha, India: a cluster-randomised trial’, *Lancet Glob. Health*, vol. 2, no. 11, pp. e645–e653, Nov. 2014, doi: 10.1016/S2214-109X(14)70307-9.

[35] S. Galiani, P. Gertler, and E. Schargrodsky, ‘Water for life: The impact of the privatization of water services on child mortality’, *J. Polit. Econ.*, vol. 113, no. 1, pp. 83–120, 2005.

[36] C. Granados and F. Sánchez, ‘Water reforms, decentralization and child mortality in Colombia, 1990–2005’, *World Dev.*, vol. 53, pp. 68–79, 2014.

[37] J. H. Humphrey *et al.*, ‘Independent and combined effects of improved water, sanitation, and hygiene, and improved complementary feeding, on child stunting and anaemia in rural Zimbabwe: a cluster-randomised trial’, *Lancet Glob. Health*, vol. 7, no. 1, pp. e132–e147, 2019.

[38] Instituto Apoyo, ‘Evaluacion de impacto y sostenibilidad de los proyectos de foncodes. Instituto Apoyo, Tegucigalpa.’ Instituto Apoyo, Tegucigalpa, 2000. Accessed: Feb. 08, 2021. [Online]. Available: https://fdocuments.ec/document/sexta-evaluacin-expost-del-foncodes-world-rubio-en-la-definicin-del-diseo.html?page=7

[39] E. Messou, S. V. Sangaré, R. Josseran, C. Le Corre, and J. Guélain, ‘[Effect of hygiene measures, water sanitation and oral rehydration therapy on diarrhea in children less than five years old in the south of Ivory Coast]’, *Bull. Soc. Pathol. Exot. 1990*, vol. 90, no. 1, pp. 44–47, 1997.

[40] A. J. Pickering, H. Djebbari, C. Lopez, M. Coulibaly, and M. L. Alzua, ‘Effect of a community-led sanitation intervention on child diarrhoea and child growth in rural Mali: a cluster-randomised controlled trial’, *Lancet Glob. Health*, vol. 3, no. 11, pp. e701–e711, Nov. 2015, doi: 10.1016/S2214-109X(15)00144-8.

[41] D. Rasella, ‘Impacto do Programa Água para Todos (PAT) sobre a morbi-mortalidade por diarreia em crianças do Estado da Bahia, Brasil’, *Cad. Saúde Pública*, vol. 29, pp. 40–50, 2003.

[42] H. Reese *et al.*, ‘Assessing longer-term effectiveness of a combined household-level piped water and sanitation intervention on child diarrhoea, acute respiratory infection, soil-transmitted helminth infection and nutritional status: a matched cohort study in rural Odisha, India’, *Int. J. Epidemiol.*, vol. 48, no. 6, pp. 1757–1767, Dec. 2019, doi: 10.1093/ije/dyz157.

[43] J. C. Semenza, L. Roberts, A. Henderson, J. Bogan, and C. H. Rubin, ‘Water distribution system and diarrheal disease transmission: a case study in Uzbekistan.’, *Am. J. Trop. Med. Hyg.*, vol. 59, no. 6, pp. 941–946, 1998.

[44] J. L. Peters, A. J. Sutton, D. R. Jones, K. R. Abrams, and L. Rushton, ‘Contour-enhanced meta-analysis funnel plots help distinguish publication bias from other causes of asymmetry’, *J. Clin. Epidemiol.*, vol. 61, no. 10, pp. 991–996, 2008.
